# Synergic Difficulties in an Anticipated Physiologically and Anatomically Difficult Airway in a Trauma Patient: A Case Report

**DOI:** 10.7759/cureus.50735

**Published:** 2023-12-18

**Authors:** Patrícia Martins Lima, Mariana Adams, Sérgio G Pinto, Carlos Mexedo

**Affiliations:** 1 Anesthesiology, Centro Hospitalar Universitário São João, Porto, PRT; 2 Anesthesiology and Critical Care, Centro Hospitalar Universitário do Porto, Porto, PRT

**Keywords:** awake intubation, difficult airway management, traumatic spinal fracture, high-flow nasal oxygen, trauma anesthesia

## Abstract

The American Society of Anesthesiologists (ASA) defines a difficult airway as a clinical situation in which a physician who is trained in anesthesiology experiences difficulty or fails in either face mask ventilation, laryngoscopy, using a supraglottic airway, tracheal intubation, extubation, or front-of-neck airway. Classically, this has been defined in relation to anatomic factors, but the concept of a physiologically difficult airway has been growing in relevance, in which physiologic factors, such as hypoxemia and hypercapnia, act to reduce safe apnea times. The case reports on a trauma patient with an unstable thoracic vertebral fracture requiring correction via the posterior approach. Our patient had multiple anatomical difficult airway predictors, namely, a short neck, greatly limited neck mobility, and a Mallampati class IV airway, among others, and multiple physiological difficult airway predictors, such as a baseline hypoxemic respiratory failure and severe sleep apnea, in addition to the restrictions on mobility imposed by the fracture itself. We describe a successful perioxygenation strategy, using high-flow nasal oxygen (HFNO) during the preoxygenation, intubation, extubation, and post-anesthesia care phases, and with an awake fiberoptic intubation technique for securing the airway.

## Introduction

The American Society of Anesthesiology (ASA) has defined a difficult airway as a clinical situation wherein an experienced physician trained in anesthesia encounters difficulty or fails to achieve success in one or more of the following: facemask ventilation, laryngoscopy, ventilation using a supraglottic airway, tracheal intubation, extubation, or front-of-neck airway. Airway assessment, through an examination of anatomical features, review of past medical history, anesthesiology records, and imaging studies, such as ultrasound and CT-scan, can help predict a difficult airway [1,2].

The incidence of a difficult airway varies according to the definition used, the population in question (age, sex, female sex, and obstetric population), and patient characteristics (e.g., obesity and ASA physical status). An incidence of 2-8% is commonly found for difficult airways [3,4].

A physiologically difficult airway represents a unique challenge distinct from an anatomically difficult airway. As explored by Fonseca et al. in their 2023 review [5], the challenges of a physiologically difficult airway are not the consequence of anatomical deviations impeding instrumented airway access, but rather physiological conditions that render the patient vulnerable to rapid desaturation, hemodynamic instability, or altered respiratory mechanics during intubation attempts. Common factors contributing to a physiologic difficult airway include severe hypoxemia, hypercarbia, acidemia, and elevated intracranial pressure. The presence of conditions, such as pulmonary shunting, reduced functional residual capacity, or an altered oxygen-hemoglobin dissociation curve, can drastically reduce safe apnea time.

Trauma patients can present additional challenges to airway management, due to swelling, airway distortion, maxillofacial injury, or spinal injuries with limited cervical mobility [6].

## Case presentation

This paper presents the case of a 72-year-old male who was proposed for a correction of a D8-9 vertebral fracture, which occurred as a consequence of a fall from his height a week prior. Throughout the week, the patient developed worsening dorsolumbar pain and loss of strength in the lower limbs, reaching a nadir of 3/5 muscle strength, limiting mobility. The patient was first admitted to a local general hospital, where a CT scan of the dorsolumbar spine was requested demonstrating a fracture of D8-D9 vertebrae and diffuse idiopathic skeletal hyperostosis. The patient was subsequently transferred to our institution, a regional teaching hospital, for surgical treatment. Upon arrival, the patient was reevaluated and found to have a decrease in muscle strength in the lower limbs, now grade 3 bilaterally. The anesthesia team on call was informed of the patient’s diagnosis and proposed surgical intervention.

A review of the patient’s chart demonstrated multiple physiological factors for a difficult airway, namely, class III obesity (with a body mass index of 44 kg/m^2^), non-stratified chronic obstructive pulmonary disease (COPD), severe sleep apnea under continuous positive airway pressure (CPAP), and nocturnal supplemental oxygen.

**Figure 1:**
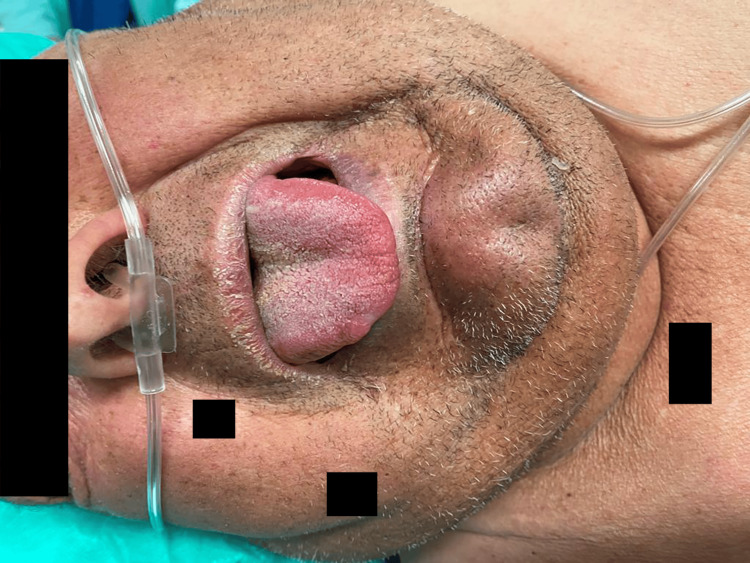
Pre-operative assessment of the patient's airway before intubation.

**Figure 2:**
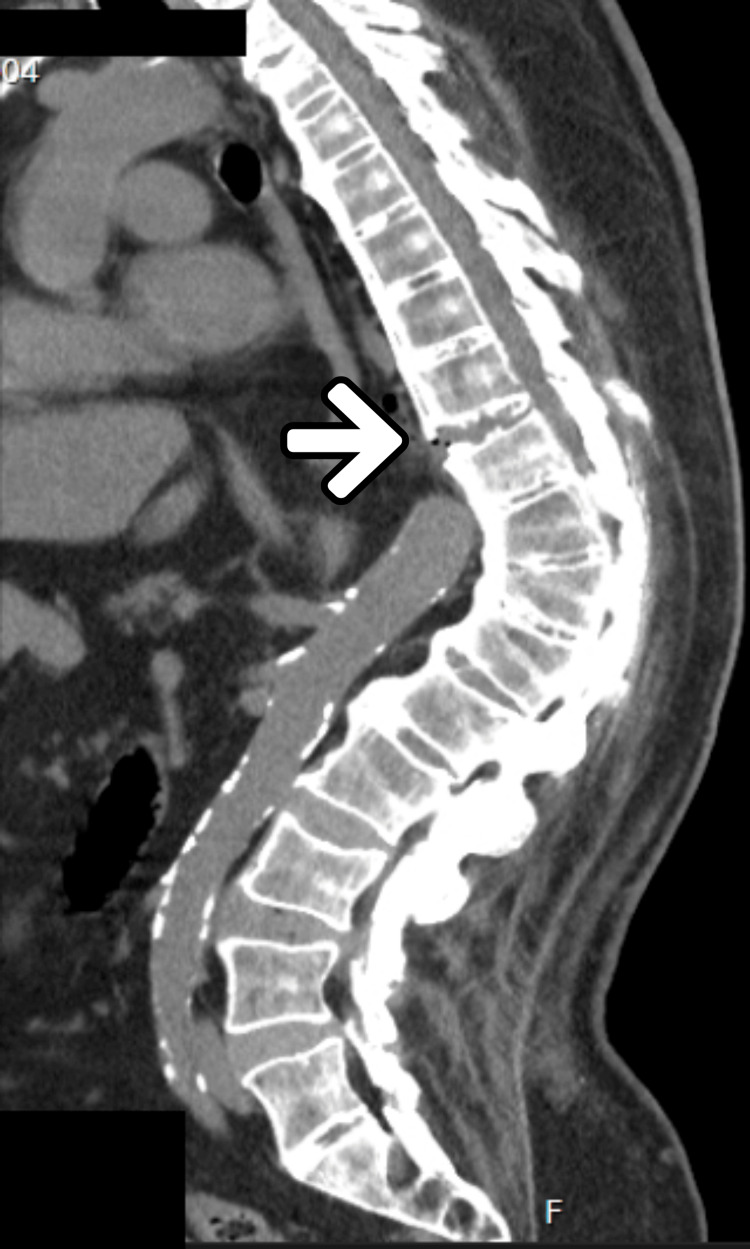
Patient's CT scan revealing important dorsal kyphosis. Arrow: D8-9 vertebral fracture, CT: computerized tomography

The preoperative assessment revealed several anatomical difficult airway stigmas, namely, a short neck with an increased circumference, limited mouth opening, reduced cervical mobility in both the lateral and anteroposterior axes, a Mallampati score of IV, an upper lip bite test class III, a thyromental distance <6 cm, and a sternomental distance <14.75 cm, as shown in Figure 1. The presence of a dorsal fracture with spinal cord compression and consequent neurological deficits, as well as the patient’s accentuated cervicodorsal kyphosis (see Figure 2), greatly hampered optimal positioning for approaching the airway, with the patient being unable to lie in the dorsal decubitus position and assuming an antalgic position of right lateral decubitus. The patient was therefore classified as having an anticipated difficult airway, and a plan of action was defined and explained to the patient and subsequently to all the members of the anesthetic team.

Preoperative laboratory findings were within the normal range, and the electrocardiogram performed showed sinus rhythm with a heart rate of 80 bpm. Upon arrival to the operating room, the patient was monitored according to the ASA Standards for Basic Anesthetic Monitoring and with invasive blood pressure via a catheter in the left radial artery, near infrared spectroscopy (NIRS) for brain oximetry, processed electroencephalogram with the bispectral index (BIS) system (Medtronic, Minneapolis, MN, USA), and hourly urinary output.

As mentioned above, the patient could not tolerate dorsal decubitus and entered the operating room in his antalgic position of right lateral decubitus. Baseline blood pressure was 171/78 mmHg, and peripheral oxygen saturation (SpO2) in ambient air was 88%. Arterial blood gas (ABG) sampling revealed respiratory acidosis and hypoxemia, with a pH of 7.30, pO_2_ of 55 mmHg, pCO_2_ of 65 mmHg, and HCO_3-_ of 32 mEq/L, suggestive of acute-on-chronic respiratory failure. Temperature and heart rate were within normal limits.

The patient was placed on a high-flow nasal oxygen (HFNO) system for pre-oxygenation with an increase of SpO_2_ values to 96-98%. To ensure patient comfort and tolerability, a ramping strategy was implemented, with an initial flow of 20 L/min for the first three minutes and then increased to 40 L/min afterward. Patient comfort was actively evaluated during this phase, with no negative feedback. Considering the patient’s comorbidities and difficult airway features, the low-level positive end-expiratory pressure (PEEP) effect HFNO imparts, and the potential wash-out of the naso- and oropharyngeal dead space was deemed beneficial. Furthermore, the comfort of the patient was taken into consideration as HFNO offers humidification with reduced air-surface dehydration and the increased tolerability of the interface as compared to the facemask.

Given the unstable nature of the patient’s vertebral fracture, minimizing unnecessary movement was paramount, and the decision was made to maintain the right lateral decubitus position during the airway approach. If necessary, the team was alert and prepared to rapidly rotate the patient to ventral decubitus, given the increased difficulty of the lateral decubitus approach. Topical anesthesia of the airway was performed with atomized lidocaine at 20 mg/ml. Adequate sedation was defined as depressed consciousness if left undisturbed but fully responsive to verbal commands. This was achieved with a remifentanil infusion in a target-controlled infusion (TCI) system using Minto's model at a 2 ng/mL effect-site target. The dose was titrated as needed to achieve conscious sedation as defined above, and once this was achieved, HNFO was increased to a flow rate of 60 L/min with no negative impact on patient tolerance.

A 4 mm intubation fiberscope (KARL STORZ, Tuttlingen, Germany) loaded with a size 7 armored endotracheal tube (ET) was passed orally to the trachea through a bite block. Under visualization of the cords and adequate fiberscope placement, the ET tube was advanced into the trachea, and general anesthesia was induced with propofol through a TCI system using Schnider’s model. The patient was stable throughout intubation, maintaining an SpO_2_ above 94%, and there were no complications. Immediately after induction, the patient suffered a brief period of hypotension, which resolved after the administration of two boluses of 100 µg of phenylephrine and adjustment of the remifentanil infusion rate.

**Figure 3:**
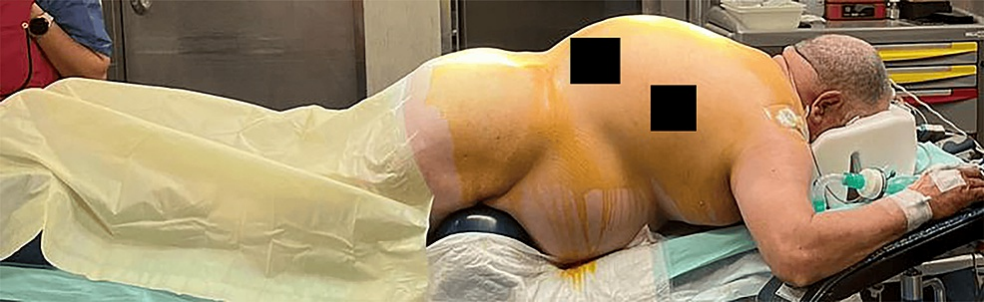
The patient's preoperative positioning, after intubation.

The patient was rotated to ventral decubitus using the logrolling technique with manual in-line stabilization. Gel cushions were used to position the patient, ensuring that the abdomen, eyes, and nose remained free of pressure and that sensitive pressure points, such as the bony prominences of the hip, were adequately protected (see Figure 3).

The surgery duration was 270 minutes. There was an estimated total blood loss of 1200 mL, and 2000 mL of polyelectrolyte solution was administered. Urinary output was normal. There were no surgical complications.

There were no anesthetic complications throughout the surgery, with an end-surgery ABG sampling revealing a pH of 7.35, a pO_2_ of 108 mmHg, and a pCO_2_ of 42.6 mmHg. The patient was extubated in the operating room and placed on a high-flow nasal cannula before transport to the post-anesthetic care unit (PACU). A multimodal approach was used for postoperative pain control, with administration of paracetamol, tramadol, lidocaine, metamizol, and ketamine. In the PACU, the patient was placed on bilevel positive airway pressure (IPAP 10 mmHg; EPAP 7 mmHg; O_2_ at 4 L/min) due to mixed respiratory and metabolic acidosis and subsequently transferred to the intensive care unit (ICU) for continuation of care. The patient remained in the ICU for 48 hours with normalization of the acid-base status and recovery of muscle strength, with no abnormalities on neurological examination noted. After 48 hours, the patient was transferred to his regional hospital for continued care.

Approximately one month after the surgery, the patient was re-evaluated in the outpatient clinic by the orthopedic surgeon and was found to have made a full recovery with no neurological deficits.

## Discussion

Traumatic spinal injuries can in and of themselves pose a great challenge to the anesthesiologist, and the patient described in this case had not only an unstable dorsal vertebral fracture but also significant comorbidities and an anticipated difficult airway due to both anatomical and physiologic factors. The significant limitations in patient positioning posed an additional difficulty.

Perioxygenation is a novel concept, describing a holistic, personalized strategy to maintain oxygenation throughout the entire anesthetic act, starting with the preoperative period and going all the way to extubation and PACU care [7].

Awake fiberoptic intubation (FOI) allows the anesthesiologist to secure the airway while maintaining spontaneous ventilation and intrinsic airway tone while also allowing precise navigation of anatomic difficulties [8]. This makes it an ideal choice for a patient like ours, with the combination of a difficult anatomy and a difficult physiology.

The authors opted to use HNFO for pre-oxygenation and supplemental oxygen therapy after extubation and in the PACU as a part of our perioxygenation strategy. HNFO has many advantages. It provides CPAP at high flow rates, decreases respiratory dead space, reduces airway resistance, and is easy to administer, and, very importantly for AFOI, it does not limit access to the airway [9]. This patient, apart from having an anatomically difficult airway, also had an anticipated physiologically difficult airway, making the use HNFO an ideal option as recommended by the Difficult Airway Society guidelines for awake tracheal intubation in adults [8].

Achieving adequate sedation while maintaining a patent airway and adequate intubating conditions is crucial for FOI. We used a remifentanil infusion via a TCI-ready infusion pump. Remifentanil is a short-acting and easily titratable synthetic opioid with a fast onset of action and rapid metabolism [10]. It is capable of suppressing airway reflexes, allowing for intubation of the trachea with a decreased need for topical anesthesia, thereby reducing the risk of local anesthetic toxicity [11].

While this case highlights a successful strategy in a patient with a constellation of anticipated airway difficulties, it is nonetheless a single-case report. With the concept of a physiologically difficult airway gaining traction in anesthesiology, it is fundamental to see further research to better define guidelines and strategies to safely broach these difficulties.

## Conclusions

Anatomic and physiologic airway difficulties act synergically to increase patient risks. The anticipation of a difficult airway allows for the formulation of a successful airway management plan that guarantees patient ventilation and oxygenation throughout the entire perioperative period.

High-flow nasal oxygen is a powerful tool to assist with intubation in challenging patients, and we used it throughout the entire perioperative period as an adjuvant to our airway approach.

Awake fiberoptic intubation remains the technique of choice for anatomically challenging airways, allowing the anesthesiologist time and safety while securing the airway.
